# Role of acute mechanical circulatory support devices in cardiogenic shock

**DOI:** 10.1007/s12055-023-01484-w

**Published:** 2023-03-28

**Authors:** Pankaj Garg, Md Walid Akram Hussain, Basar Sareyyupoglu

**Affiliations:** 1 Department of Cardiothoracic Surgery, Mayo Clinic, 4500 San Pablo Road, Jacksonville, FL 32224 USA; 2 Cardiothoracic Surgery, Heart and Lung Transplant Program, Mayo Clinic, 4500 San Pablo Road, FL 32224 Jacksonville, USA

**Keywords:** Cardiogenic shock, Acute mechanical circulatory support (AMCS) devices, Low cardiac output

## Abstract

Cardiogenic shock is a state of low cardiac output that is associated with significant morbidity and mortality. A considerable proportion of patients with cardiogenic shock respond poorly to medical management and require acute mechanical circulatory support (AMCS) devices to improve tissue perfusion as well as to support the heart. In the last two decades, many new AMCS devices have been introduced to support the right, left, and both ventricles. All these devices vary in terms of the support they provide to the body and heart, mechanism of functioning, method of insertion, and adverse events. In this review, we compare and contrast the available percutaneous and surgically placed AMCS devices used in cardiogenic shock and discuss the associated clinical and hemodynamic data to make a conscious decision about choosing a device.

## Introduction

Cardiogenic shock (CS) is defined as “a state of low cardiac output (LCO) associated with hypotension and evidence of end-organ hypoperfusion.” Hemodynamic criteria include systolic blood pressure (SBP) < 90 mmHg, cardiac index (CI) ≤ 2.0–2.2 L/min/m^2^, and elevated pulmonary capillary wedge pressure (PCWP) > 15 mmHg [1]. This definition tends to keep all the patients with CS under a single umbrella. This has not only created confusion in interpreting the results of various studies but also failed to show any survival benefit with the use of any acute mechanical circulatory support (AMCS) device despite the contrary is observed in the clinical setting. Patients with CS in reality are quite diverse in their hemodynamic, metabolic, and organ function derangement and so in their clinical outcomes [2]. Furthermore, CS can be due to left heart failure (LHF) or right heart failure (RHF), or biventricular failure. In 2019, the Society of Cardiovascular Angiography and Intervention (SCAI) proposed a dynamic classification of CS and further modified it in 2022. It classified CS into five stages based on clinical, hemodynamic, and metabolic severity. Among them, stages A and B are prior to the onset of clinically overt CS. Recognizing patients at this stage and acting appropriately can prevent the slipping of many patients into stages C to E and may improve their outcomes [3] (Table 1).

**Table 1:** The Society of Cardiovascular Angiography and Interventions stages of cardiogenic shock

Stage of shock	Physical examination	Hemodynamics	Biochemical markers	Description
Stage A At risk	Normal physical exam	Normotensive CI ≥ 2.5 L/min/m^2^ CVP < 10 mmHg PA saturation ≥ 65%	Normal renal function Lactate < 2 mmol/L	Patients not in CS, but are at risk of developing CS (patients with STEMI, NSTEMI, acute or acute-on-chronic CHF)
Stage B Beginning	Elevated JVP Rales in lung fields No signs of peripheral hypoperfusion	SBP < 90 or MAP < 60 or > 30 mmHg drop from baseline Pulse ≥ 100 bpm CI ≥ 2.2 L/min/m^2^ CVP > 12 mmHg PA sat ≥ 65%	Normal/ minimally impaired renal function Lactate < 2 mmol/L Elevated BNP	Clinical evidence of hypotension or tachycardia without hypoperfusion
Stage C Classic	Patient looks unwell Skin ashen cold, clammy, mottled, dusky Volume overload Rales throughout lung fields Killip class 3 or 4 BiPAP or mechanical ventilation Acutely altered mental status Urine output < 30 mL/h	SBP < 90; MAP < 60 or > 30 mmHg drop from baseline and drugs/device to maintain BP above these targets CI < 2.2 L/min/m^2^ PCWP > 15 mmHg CVP/PCWP ≥ 0.8 PAPi < 1.85 CPO ≤ 0.6 W/m^2^	Lactate ≥ 2 mmol/L: serum creatinine doubling or > 50% drop in GFR; elevated LFTs and BNP	Clinical evidence of hypoperfusion requiring medications/MCS beyond volume resuscitation to restore perfusion
Stage D Deteriorating/doom		Stage C + requiring multiple pressors or MCS devices to maintain perfusion	Stage C and deteriorating	Similar to stage C, but getting worse and failed to respond to initial interventions
Stage E Extremis	Near pulseless Cardiac collapse Mechanical ventilation Defibrillator used	Hypotensive despite maximal support	pH ≤ 7.2 Lactate ≥ 5 mmol/L	Cardiac arrest (PEA or refractory VT/VF) with ongoing CPR or ECLS placement

*CI*, cardiac index; *CVP*, central venous pressure; *PA*, pulmonary artery; *CS*, cardiogenic shock; *NSTEMI*, non-ST elevation myocardial infarction; *CHF*, congestive heart failure; *SBP*, systolic blood pressure; *MAP*, mean arterial pressure; *BNP*, B-type natriuretic peptide; *bpm*, beats per minute; *BiPAP*, bilevel positive airway pressure; *PAPi*, pulmonary artery pulsatility index; *PCWP*, pulmonary capillary wedge pressure; *CPO*, cardiac power output; *GFR*, glomerular filtration rate; *LFT*, liver function test; *MCS*, mechanical circulatory support; *CPR*, cardiopulmonary resuscitation; *ECLS*, extracorporeal life support; *PEA*, pulseless electrical activity; *sat*, saturation; *VF*, ventricular fibrillation; *VT*, ventricular tachycardia

**Table 2:** Potential indications for acute mechanical circulatory support device in cardiogenic shock

Acute myocardial infarction
Mechanical complications related to acute myocardial infarction • Ventricular septal defect • Ischemic mitral regurgitation
Acute on chronic heart failure
Acute right ventricular failure • Right ventricular myocardial infarction • Right ventricular failure after left ventricular assist device
Acute myocarditis
Refractory cardiac arrest
Refractory arrhythmias
Post-cardiotomy shock
Severe valvular heart disease
Post-heart transplant primary graft dysfunction
Stress cardiomyopathy (Takotsubo cardiomyopathy)
Peripartum cardiomyopathy
Cardiac trauma with or without cardiac tamponade
Acute pulmonary embolism
Severe pulmonary hypertension
Drug intoxication
High-risk interventions • Percutaneous coronary intervention • Ventricular tachycardia ablation

## Etiology of cardiogenic shock

Acute myocardial infarction (AMI) remains the leading cause of CS. However, non–AMI-related CS are on the rise. Etiologies of cardiogenic shock are enumerated in Table 2 [4]. The management and response to therapeutic interventions for each of these scenarios vary greatly. Unfortunately, CS continues to be associated with significant morbidity and 30 to 60% mortality despite improvements in medical management and use of AMCS devices [5]. Intra-aortic balloon pump (IABP) was the first AMCS device introduced five decades back. Since then, several AMCS devices have been introduced including Impella, Protec Duo cannula, CentriMag pump, TandemHeart, and veno-arterial extracorporeal membrane oxygenator (VA-ECMO) to provide left- and/or right-heart support [6]. Among all AMCS devices, IABP remains the most widely used, although the use of other more robust devices is increasing [6–8]. Indiscriminate use of AMCS devices without improvement in patient outcomes has brought disrepute to many of these devices. However, understanding the pathogenesis and severity of CS and the functioning of each AMCS device with its strengths and shortcomings can lead to a conscious selection of the device and improve the patient outcome. Furthermore, two or more devices can be combined to overcome the shortcoming of each device and achieve the desired support.

Early identification and timely initiation of optimal medical therapy (OMT) are the mainstay of managing the patients with CS SCAI stage A and B. Patients if optimally managed early enough can be prevented to slip into CS stage C to E. However, if the patient slumps down the spiral of CS or already in advanced CS at presentation, he should be managed with an AMCS device. Before choosing an AMCS device, it is important to answer the following questions.

**Fig. 1:**
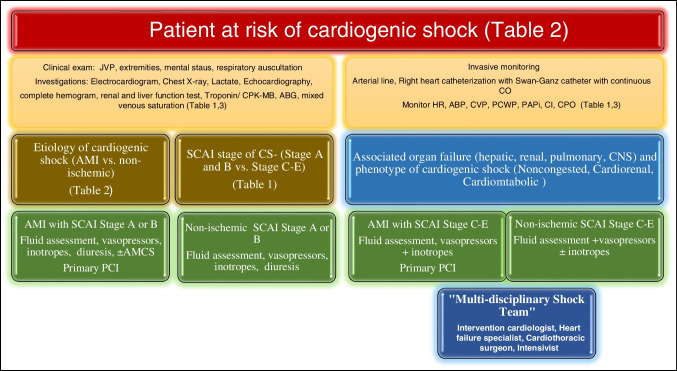
Diagnosis and management of cardiogenic shock

1. Type of heart failure: LHF, RHF, biventricular failure (Fig. 1 and Table 3)

**Table 3:**
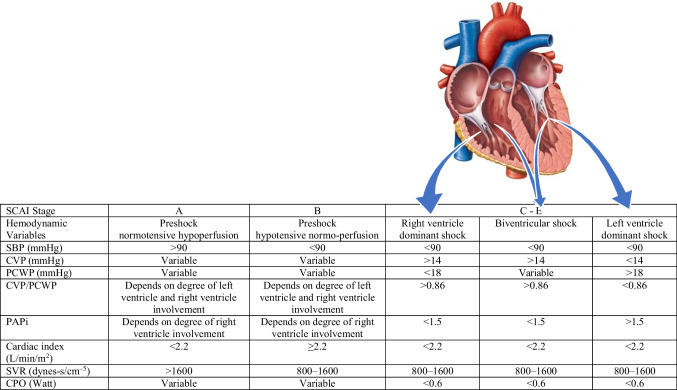
Hemodynamics parameters in SCAI Stage A to E and predominant right, left, and biventricular failure

*SBP*, systolic blood pressure; *CPO*, cardiac power output; *CVP*, central venous pressure; *PAPi*, pulmonary artery pulsatility index; *PCWP*, pulmonary capillary wedge pressure; *SVR*, systemic vascular resistance

2. Etiology of CS (Table 2)

3. Stage of cardiogenic shock (SCAI staging A to E) (Table 1) [3]

4. Associated metabolic profile (organ failure), i.e., non-congested, cardiorenal, cardiometabolic [5]

5. History of cardiac arrest with anoxic brain injury (SCAI Update) [3]

6. Goal of using an AMCS device. These goals can be in isolation or in combination and include improving organ perfusion, improving coronary blood flow (CBF), unloading the left ventricle (LV) and/or right ventricle (RV), and reducing pulmonary artery pressure (PAP), reducing shunting across ventricular septal defect (VSD), and reducing mitral regurgitation (MR)

**Fig. 2:**
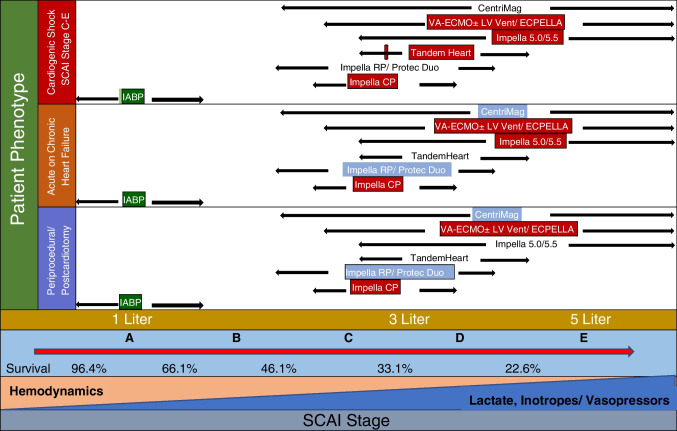
Choice of the right MCS should be guided by patient phenotype, hemodynamic needs, risk of complication, and AMCS availability. IABP is marked in green suggesting that it can be AMCS in any patient until further decision is made. Typical needs for patients based on phenotype are highlighted in red for left heart and blue for right heart. Most patients usually require peri-procedural AMCS for 1–3 L/min of support, whereas patients presenting in cardiogenic shock typically require 3–5 L/min of support. AMCS should be chosen accordingly. Patients with decompensated HF often can be treated quickly with medical therapy or an IABP; however, in patients with significant end-organ hypoperfusion, complete hemodynamic support is often needed

To achieve these goals appropriately, whenever feasible, we should do a complete transthoracic (TTE) or transesophageal echocardiography (TEE), the right heart catheterization with pulmonary artery wedging using a Swan-Ganz catheter to assess the baseline left and right heart pressure and hemodynamic data including central venous pressure (CVP), PCWP, and CI (Fig. 2). Furthermore, every hospital should make their “Shock Team” that should comprise an intervention cardiologist, heart failure specialist, intensivist, cardiac surgeon, cardiac anesthetist, and any other stakeholder. The team should be available 24/7 to brainstorm about the best management strategy and plan of care including referral and withdrawal of care for any patient with CS. The decision should be made with the consensus keeping the ultimate goal of care, especially when selecting an AMCS device for the management of CS.

In this review, we highlight the available AMCS devices, their strengths and weaknesses, the associated clinical and hemodynamic data, and the algorithm for decision making.

## Acute mechanical circulatory support devices

The available AMCS devices used to treat CS are listed in Table 4 while indications for use and complications are listed in Table 5. These devices are often used as a bridge either to recovery, or bridge to transplant or as a durable ventricular assist device.

**Table 4:** Technical features and hemodynamic effects of acute mechanical circulatory support devices

	Access	Access sites Artery	Access sites Vein	Inflow	Outflow	Cannula size (Fr.)	Flow range, L/min	RAP, mm Hg	Mean PAP, mm Hg	PCWP or LVEDP, mm Hg	LV afterload (MAP)	Native CO	Coronary perfusion	Myocardial oxygen demand	Respiratory support
Isolated RHF AMCS devices															
Impella RP	PC	–	FV	RA	PA	23	2–4	↓	↑	↑↑	↔	↔ ↑	↔	↑	–
Protek Duo	PC	–	IJV	RA	PA	29	2–4	↓	↑	↑↑	↔	↔ ↑	↔	↑	–
CentriMag	Sur	PA	RA, FV	RA	PA	Arterial 15–19 Venous 18–29	2–9.9	↓	↑	↑↑	↔	↔ ↑	↔	↑	
VA-ECMO	PC/ Sur	FA, AA, SCA, Ascending aorta	FV, IJV, RA	RA	FA	Arterial 15–19 Venous 18–29	2–6	↓	↔ ↓	↓	↑↑	↔ ↓	↔	↔ ↑	+
Isolated LHF AMCS devices															
Intra-aortic balloon pump	PC/Sur	FA, AA, SCA	–	DTA	DTA	7–8	–	↔	↓	↓	↓	↑	↑	↓	–
Impella CP/5.0/5.5	PC/Sur	FA, AA, SCA	–	LV	Ao	13–18	2–5.5	↔	↓	↓↓	↓	↑	↑↑	↓↓	–
TandemHeart	PC	FA	FV			Arterial 15–19 Venous 21	2–3.5						↑↑	↓↓	
Biventricular support devices															
Impella CP/5.0/5.5 + Impella RP	PC/Sur	FA, SCA, AA	–	LV	Ao	13–18	2–5.5	↓	↑	↔ ↓↓	↔ ↓	↓	↑↑	↓↓	–
		–	FV	RA	PA	23									
VA-ECMO + LV vent	Sur	FA, AA, SCA, Ascending aorta	FV/RA/IJV	RA	Ao	Arterial 15–19 Venous 18–29	2–6	↓↓	↓	↔ ↓↓	↑↑	↓	↑	↓	+
		–	RSPV, PA, LV apex	LV	Venous cannula	14–18									
ECPELLA	Sur ± PC	FA, SCA, AA	FV/RA/IJV	RA	Ao	Arterial 15–19 Venous 18–29	2–6	↓↓	↓↓	↓↓	↔ ↓↓	↓↓	↑↑	↓	+
		FA, SCA, AA	–	LV	Ao	13–18									
VA-ECMO + LV CentriMag	Sur ± PC	FA, SCA, AA	FV/RA/IJV	RA	Ao	Arterial 15–19 Venous 18–29	2–6	↓↓	↓↓	↓↓↓	↑↑	↓↓	↑	↓	+
		–	LV apex	LV	Venous cannula	Variable									
Impella CP/5.0/5.5 + Protec Duo	PC/Sur	FA, AA, SCA	–	LV	Ao	13–18	2–5.5	↓	↑↑	↔ ↓↓	↓	↔ ↑	↑↑	↓↓	–
		–	IJV	RA	PA	29									

*RHF*, right heart failure; *AMCS*, acute mechanical circulatory support; *RPM*, rotation per minute; *PC*, percutaneous; *Sur*, Surgical; *FV*, femoral vein; *Ao*, aorta; *CO*, cardiac output; *FA*, femoral artery; *PA*, pulmonary artery; *AA*, axillary artery; *SCA*, subclavian artery; *LV*, left ventricle; *LVEDP*, left ventricular end-diastolic pressure; *MAP*, mean arterial pressure; *PA*, pulmonary artery; *PAP*, pulmonary artery pressure; *RSPV*, right superior pulmonary vein; *PCWP*, pulmonary capillary wedge pressure; *RA*, right atrial; *RAP*, right atrial pressure; *RV*, right ventricular; *DTA*, descending aorta; *RVAD*, right ventricular assist device; *VA-ECMO*, venoarterial extracorporeal membrane oxygenation; ↔ , no to minimal change; ↔ ↑, no change or mild increase; ↔ ↓, no change or mild decrease

**Table 5:** Indications and clinical considerations for various AMCS devices

	Intra-aortic balloon pump	Impella	TandemHeart	Protec Duo	CentriMag	VA-ECMO
Indications	Initial AMCS in CS, CS SCAI Stage A and B, ACHF, high risk PCI, AMI with < TIMI 0–1 flow post-PCI, post-cardiotomy shock	ACHF, cardiogenic shock SCAI stage C-E, refractory malignant arrhythmias, high-risk PCI, LV venting after VA-ECMO	ACHF, cardiogenic shock SCAI stage C-E, refractory malignant arrhythmias, high-risk PCI, LV venting after VA-ECMO	RVMI, Post cardiotomy RV failure, post LVAD and post Impella acute RV failure	RVMI, Post cardiotomy RV failure, post-LVAD and post-Impella acute RV failure	ACHF, CS SCAI stage C-E, associated respiratory failure, massive PE, cardiac arrest, refractory malignant arrhythmias
Support provided	Minimal hemodynamic support (0.5–1 L)	2.5 and CP: partial LV support 5.0, 5.5: complete LV support	Partial to complete RV support depending upon size of cannula	Complete RV support (4.0 L)	Partial to complete LV support based upon the size of the arterial cannula	Predominant RV support, with relatively preserved LV function or with LV venting provides complete biventricular support
Considerations	Readily available, minimal or no anticoagulation, patient can be ambulant with axillary or subclavian artery IABP	Active LV unloading; surgical axillary or subclavian artery placement over chimney graft allows for mobility and prolonged support	Technically demanding as requires septostomy, indirect LV unloading by decompressing the left atrium, patient is usually not or minimally ambulant	Active RV unloading; percutaneously placed through IJV. Allow mobility and prolonged support	Active RV unloading; classically, surgically placed through sternotomy or thoracotomy, Allow mobility and prolonged support	Field and bedside insertion is possible, may require strategies to unload the LV. Ideal for active cardiac arrest by providing complete cardiac and pulmonary support, labor intensive (nursing/ perfusionist)
Management	CXR for position (tip 1–4 cm below AO notch), wean by ↓ ratio from 1:1 to 1:3	CXR daily/echo for position, flow vary from P1 (lowest) to P9 (highest), hemolysis problem with CP and 2.5, ventricular arrhythmias with 5.0 and 5.5, suction events (↓ preload, RV failure, position)	Chest x ray and echo to ensure daily optimal device placement due to risk of migration from LA to RA, avoid patient maneuvering	CXR daily for position, can be managed with minimal anticoagulation, minimal risk of position change to RV	CXR daily for position, can be managed with minimal anticoagulation, usually stable position	Usually stable position, monitor for north/south syndrome and LV distension
Complications	Limb ischemia, vascular injury, thromboembolism, bleeding, stroke, balloon leak/rupture	Bleeding, limb ischemia, thromboembolism, hemolysis, vascular injury, stroke	Bleeding, limb ischemia, thromboembolism, vascular injury, stroke, residual atrial septal defect	Bleeding, thrombosis, vascular injury, PA or cardiac chamber perforation	Bleeding, thrombosis, vascular injury	Bleeding, limb ischemia, thromboembolism, vascular injury, stroke
Anticoagulation	Low	Low	High	Low	Low	high
Resource and work-force requirement	Low	Low	Medium	Low	Low	High

*AMCS*, acute mechanical circulatory support; *CS*, cardiogenic shock; *SCAI*, Society of Cardiovascular Angiography and Interventions; *ACHF*, acute on chronic heart failure; *PCI*, percutaneous coronary intervention; *PCWP*, pulmonary capillary wedge pressure; *PE*, pulmonary embolism; *RA*, right atrium; *RV*, right ventricle; *TIMI*, thrombolysis in MI; *VA-ECMO*, veno-arterial extracorporeal membrane oxygenation

## AMCS devices for left ventricular support

### Intra-aortic balloon pump

The intra-aortic balloon pump (IABP) works on the principle of counterpulsation with inflation during diastole and deflation during systole synchronized with either electrocardiogram (ECG), pressure trigger, or internal at a fixed rate. Femoral artery (FA) is the preferred site for access due to the ease of insertion. However, it limits the patient ambulation and has risk of bleeding and kinking. On the other hand, axillary artery (AxA) and subclavian artery (SCA) access allows the patient ambulation, has a lower infection rate, and provides access for prolonged duration (acute on chronic heart failure (ACHF) patients awaiting heart transplant) [9]. In our institute, we place the IABP through the AxA over chimney graft in patients with ischemic cardiomyopathy awaiting heart transplant.

#### Physiological and hemodynamic effects of IABP

The IABP results in decreased left ventricular end-diastolic pressure (LVEDP), myocardial oxygen demand, 0.5- to 1-L increase in cardiac output, and augmentation of CBF. Compared to the other available AMCS devices, support provided by IABP is the least [10].

#### Clinical data on use of IABP

Animal and human studies around the turn of century revealed the beneficial effect of IABP in improving coronary and corporeal perfusion and reducing the myocardial oxygen demand in the setting of HF [11, 12]. The SHOCK (Should We Emergently Revascularize Occluded Coronaries for Cardiogenic Shock) registry also revealed > 15% reduction in in-hospital mortality with use of IABP in patients with AMI treated by thrombolysis (46.5% in thrombolysis and IABP group vs 62.9% in thrombolysis alone group, *P* < 0.005). This led to widespread use of IABP in patients with AMI. Subsequently, SHOCK trial and registry established the role of early revascularization with percutaneous coronary intervention (PCI) or coronary artery bypass grafting (CABG) on the survival [13, 14] and IABP-SHOCK II (Intraaortic Balloon Support for Myocardial Infarction with Cardiogenic Shock) trial failed to demonstrate benefit of IABP over medical therapy in reducing 30-day, 12-month, and 6-year mortality or secondary outcome in patients with AMI immediately prior to PCI or CABG [15–17]. Therefore, IABP use in patients with AMI-CS was downgraded to class IIIB in the European Society of Cardiology and class IIb in the American College of Cardiology/American Heart Association guidelines [18, 19]. However, the SHOCK-IABP II study had several limitations [20] and recent studies including subgroup analysis of Counterpulsation to Reduce Infarct Size Pre-PCI Acute Myocardial Infarction (CRISP-AMI trial) have shown mortality benefit of IABP in patients with final TIMI 0/1 flow or no-reflow after PCI despite recurrent AMI and lower left ventricle ejection fraction (LVEF) [21–23].

#### Clinical applications of IABP

IABP can provide sufficient support for patients in SCAI stage A and B with unstable angina and ongoing ischemia, patients undergoing CABG with or without mitral valve (MV) repair with left ventricle ejection fraction (LVEF) > 35% and left ventricle end-diastolic pressure (LVEDP) < 20 mmHg, and patients with ACHF with LVEDP < 20 mmHg and mild to moderate pulmonary hypertension (PHT) awaiting heart transplantation. Use of IABP in these patients improves the hemodynamic and metabolic profile as well as improves the United Network for Organ Sharing (UNOS) waitlist status. Recently, European Society of Cardiology has also recommended IABP use for (1) patients with CS SCAI stage A and B due to AMI or non-ischemic etiology with mean arterial pressure (MAP) > 65–70 mmHg and/or mixed venous saturation (SvO_2_) < 65–70% and/or increased lactate and/or cardiac index 2–2.2 L/min/m^2^ with only one vasopressor/inotrope at low dosage; (2) patient at high risk of developing CS (signs of pulmonary congestion, no response to pharmacological therapy (especially diuretics), oliguria, tachycardia); (3) patients with persistent ischaemia/no-reflow after PCI with intractable angina or HF, and LV thrombus. In these patients, IABP is used either as bridge to recovery or bridge to escalation to more potent AMCS devices or left ventricular assist device (LVAD) placement/transplantation (bridge to decision); (4) patients with AMI-CS SCAI Stage C having mechanical complications including acute ischemic MR, VSD as bridge to surgery; (5) high-risk postcardiotomy patients, to reduce peri-procedural complications and to facilitate weaning from cardiopulmonary bypass (CPB), e.g., LCO after CABG, valvular heart surgery; (6) patients with refractory ventricular arrhythmias as a “bridge to recovery” or “bridge to treatment” (ablation, LVAD, transplantation); (7) LV unloading in patients undergoing VA-ECMO [20].

## Impella devices

The Impella device (Impella, Abiomed, Danvers, MA) is a percutaneous transvalvular microaxial flow pump that works on the principle of a “rotating Archimedes screw within a hollow pipe.” It pumps blood either from the LV to the ascending aorta by traversing the aortic valve (AoV) or from the inferior vena cava to the pulmonary artery by traversing the tricuspid and pulmonary valve. Impella devices available for left heart support are Impella 2.5 (2.5 L/min), Impella Cardiac Power (CP and CP SmartAssist; 3.5 L/min and 4.3 L/min), and Impella 5.0 and 5.5 Smart Assist (5.0 L/min and 5.5 L/min)) and for right heart support is Impella RP® (4.0 L/min). With “SmartAssist” fiberoptic pressure sensor technology in newer devices, Impella monitor can display the real-time hemodynamic metrics including LVDEP, MAP, and cardiac power output (CPO) that enable optimized positioning, managing, and weaning of the Impella [24].

Impella device is placed percutaneously or surgically under combined fluoroscopic and echocardiographic guidance. Impella 2.5 and Impella CP can be placed percutaneously via FA, while Impella 5.0 and Impella 5.5 are placed surgically through FA, SCA, or AxA over a chimney graft. Impella RP® is inserted through the femoral vein [25]. Similar to IABP, FA access restricts the patient ambulation and is good for short-term use while AxA or SCA access can be kept for a longer duration without limiting the patient ambulation. In our institute, we insert Impella 5.5 over chimney graft through AxA for patients with ACHF with CS as a bridge to transplant or durable LVAD, while we place Impella CP through FA in patients with AMI-CS requiring support for a short duration.

### Physiological and hemodynamic effects of Impella device

The Impella device improves MAP, cardiac output, organ perfusion, and CBF by pumping the blood from the LV into the ascending aorta. Impella also enables direct unloading of LV with reduced left ventricle end-diastolic volume (LVEDV), LVEDP, LV stroke work, and myocardial oxygen demand, decrease in PAP, and secondary reduction in RV afterload. Decrease in LV stroke work is highlighted by conversion of pressure–volume loop into a triangular shape due to progressive loss of isovolumic contraction phase. Improvement in CBF occurs even in ischemic territory due to concomitant increase in MAP and decreased myocardial wall tension [26]. In patients with AMI-CS, improved CBF is seen with the Impella device even prior to angioplasty and it improves the heart’s ability to survive ischemic challenges [24, 27].

### Contraindications

LV thrombus and mechanical AoV are absolute contraindications while severe AR, aortic stenosis (AS), small LV cavity such as hypertrophic obstructive cardiomyopathy and cardiac amyloidosis, post-AMI VSD, hemolytic preponderance, ascending aortic aneurysm, and severe peripheral arterial disease (PAD) are relative contraindications.

### Complications

Limb ischemia (0.07–10%), vascular injury and bleeding (0.05 to 54%), hemolysis and hemolysis induced acute renal failure (5–10%). Device migration may result in ventricular arrhythmias or rarely LV perforation/tamponade, acute mitral or aortic valve regurgitation, and thrombotic complications. Stroke may occur in 2.4 to 6.3% patients [24].

### Current evidence

Large registries have shown the safety and feasibility of the Impella 2.5 and CP devices in patients with AMI-CS (6). However, ISAR-SHOCK (The Efficacy Study of LV Assist Device to Treat Patients With Cardiogenic Shock), PROTECT II (protected PCI), and IMPRESS (Impella Versus IABP Reduces Mortality in STEMI Patients Treated With Primary PCI in Severe Cardiogenic Shock) trials failed to show any survival benefit with Impella over IABP. All these trials, however, were underpowered, included critically ill patients and the major cause of death of the patients was anoxic brain injury [6, 28, 29]. A recent study has shown that the initiation of Impella prior to PCI in patients with AMI-CS without cardiac arrest may improve outcome [25, 30, 31].

### Clinical application of Impella: ventricular unloading

Ventricular unloading refers to reduction in ventricular pressure and volume by actively aspirating the blood from the ventricle. Timely implantation of Impella devices in patients with CS or at risk of CS can prevent the progression of these patients to an irreversible shock spiral. Patients with CS due to AMI, post-cardiotomy LV failure, fulminant myocarditis, ACHF, and severe AS with severe LV dysfunction require emergency ventricular unloading, while patients undergoing high-risk PCI, catheter ablations of ventricular tachycardia, and high-risk CABG may benefit from preemptive ventricular unloading [19].

Post-cardiotomy CS occurs in 0.2–9% of patients and has high mortality. The study involving 24 patients with post-cardiotomy CS who were managed with an Impella device showed significantly decreased mortality compared to IABP if the heart had residual cardiac contractility reserve to pump > 1 L/min blood [26]. A prophylactic Impella device may also be useful in undergoing emergent CABG for AMI-CS and off-pump CABG to minimize cardiovascular instability during the procedure. Despite these promising reports, VA-ECMO is still more commonly employed in patients with post-cardiotomy CS and further studies are necessary to explore the role of Impella device in this setting [19].

## TandemHeart system

The TandemHeart system (Cardiac Assist Inc, Pittsburgh, PA) is a percutaneously placed centrifugal temporary LVAD that works on the concept of left heart bypass (LHB) and unloads the LV by actively withdrawing the oxygenated blood directly from the left atrium and pumping directly into the distal descending aorta [32].

### Physiological and hemodynamic effects of TandemHeart

Similar to Impella, the TandemHeart device reduces LVEDV, LVEDP, and stroke work. Hemodynamically, the TandemHeart significantly reduces LV preload and augments cardiac output, with the ability to pump up to 5.0 L/min blood [11, 12]. Importantly, the TandemHeart is preload dependent and works optimally at PCWP of 18 to 20 mmHg. However, similar to extracorporeal membrane oxygenator (ECMO), the TandemHeart significantly increases the afterload from retrograde aortic blood flow offsetting some benefit of reduced myocardial oxygen demand by LV unloading [32].

### TandemHeart clinical data

TandemHeart was introduced in 2006. Initial enthusiasm about the device the device quickly waned due to the complexity of its insertion and higher incidence of complications. Randomized studies comparing TandemHeart with IABP in 2005 and 2006 found not only superior hemodynamics with TandemHeart, i.e., higher CPO and lower PCWP, but also more complications [6, 33]. After the success of Impella devices, TandemHeart is scarcely used now.

## Devices for acute right heart failure and right ventricular unloading in cardiogenic shock

Acute RHF can develop primarily due to decreased RV contractility (e.g., right ventricle MI (RVMI), myocarditis, post-cardiotomy, or post LVAD support) or secondary to increased RV afterload (e.g., acute pulmonary embolus, severe hypoxia, acidemia, or raised intrathoracic pressures). Although, due to the ventricular interdependence, it is difficult to define acute primary RHF, still, it can be diagnosed with reasonable certainty in the presence of CI < 2.0–2.2 L/min/m^2^ and CVP > 15 mmHg, PCWP < 15 mmHg, and PA systolic pressure < 50 mmHg. In the presence of PCWP > 15 mmHg, RHF is secondary to LHF, while transpulmonary gradient < 15 mmHg and systolic PAP > 50 mmHg point toward pulmonary etiology. Echocardiographically, there is the presence of moderate to severe global RV dysfunction, tricuspid annular plane systolic excursion (TAPSE) ≤ 14 mm, RV diameter at base > 42 mm, and mid-cavity > 35 mm [2, 27].

### Impella RP

Impella RP is an AMCS device for RV support that may serve as a bridge to recovery or transplant. Since survival after Impella RP insertion strongly depends on timing and patient selection, early identification of RHF and careful consideration of patient’s clinical status and comorbidities are key to best clinical outcomes [22, 27, 34, 35]. The Impella RP device is inserted percutaneously through the femoral vein under combined fluoroscopic and echocardiographic guidance [25].

#### Hemodynamic effects and evidence

Like other Impella devices, Impella RP unloads the RV with reduction in right ventricular end diastolic pressure (RVEDP), right ventricular end-diastolic volume (RVEDV), RV stroke work, myocardial oxygen demand, and CVP while maintaining the LV preload. All these improve the effective organ perfusion [26]. In a recent RECOVER RIGHT trial, Anderson et al. demonstrated the feasibility, safety, and reliability of Impella RP to improve hemodynamics in select patients with refractory acute RHF. In 30 patients with acute RHF, 18 patients had RHF after LVAD implantation, and 12 patients after cardiotomy or AMI. Thirty-day survival was 73.3%, and all discharged patients were alive at 6 months. Similar findings have been reported by other recent studies with 64–72% 30-day survival. All these studies reported when Impella RP was implanted as salvage support after prolonged CS (> 48 h) and in-hospital cardiac arrest emphasizing the need for proper patient selection and early initiation of hemodynamic support [34, 36–38].

### Protek Duo system

Protek Duo (TandemLife, Pittsburgh, PA, USA) is a double lumen cannula that was introduced in 2014 for temporary right heart support. Protek Duo 29 Fr. cannula is inserted percutaneously under fluoroscopic guidance through the right internal jugular vein (RIJV) with distal limb placed into the main pulmonary artery (MPA) and proximal holes in RA. A single cannula in the neck permits simultaneous drainage of blood from the RA and reinfusion into the PA and improved patient ambulation, and the cannula can be removed at the bedside under sedation. Like Impella RP, Protek Duo also reduces RVEDP, RVEDV, RV stroke work, myocardial oxygen demand, and CVP.

Protek Duo cannula is useful for acute RHF with CS and normal functioning lungs. However, in contrast to Impella RP, the Protek Duo cannula can also be used in patients with acute RHF with acute respiratory distress syndrome (ARDS) by slicing the oxygenator into the circuit. This is called oxy-right ventricular assist device (oxy-RVAD). Furthermore, the need for anticoagulation is less and it does not increase LV afterload while maintaining the preload.

In recently published observational studies, improved outcomes were observed for patients with RHF who underwent timely placement of Protek Duo [39, 40]. In a retrospective series by Usman et al. including 24 patients with acute RHF, survival with protek Duo device was 66.6%. Authors found that the survivors had lower vasopressor-Inotrope score (VIS) scores at the time of insertion and decannulation and lower CVP at the time of decannulation [41]. One of the limitations of Protek Duo system is that the cannula (29 F) occupies a large portion of the right ventricular outflow tract and PA; therefore, standard continuous hemodynamic monitoring with a pulmonary artery catheter is commonly not used. Serial echocardiography is pivotal for device deployment, monitoring device position, assessing RV readiness for decannulation, and surveilling for short-term complications [41].

### CentriMag

CentriMag (Abbott Laboratories, Abbott Park, IL, USA) was the first magnetically levitated pump introduced as a temporary ventricular assist device (VAD) in 2003 and since then, more than 40,000 devices have been implanted and > 150 case series and reports have published their results. Presently available CentriMag is the second-generation system and includes a reusable motor, a centrifugal pump, a flow probe, and a console. The system can be connected to surgically or percutaneously placed central or peripheral cannula, can be combined with other AMCS devices such as Protek Duo cannula, and the oxygenator can also be sliced into the circuit to convert it to oxy-VAD. Due to the magnetic levitation technology, the pump operates without mechanical bearings or seals and provide up to 10 L/min of flow without significant turbulence, wear, friction, heat generation, and blood trauma even on prolonged use [42].

#### Indications

CentriMag functions as a robust temporary VAD that can be used for short‑term cardiopulmonary support (up to 30 days) in patients with CS due to AMI, ACHF, post-heart transplant primary graft dysfunction, post-cardiotomy, and acute RHF. It can be used as a bridge to decision, bridge to recovery, or bridge to transplant. The device can provide either left or right ventricular support (LVAD or RVAD) or total circulatory support (biventricular assist device (BiVAD)). It can also be used as a part of ECMO circuit by slicing an oxygenator in the circuit [42–45]. In a large study by Mehta and Venkateswaran from the UK involving sixty-three patients who underwent extracorporeal membrane oxygenation (ECMO) using CentriMag over a period over 12 years (2005–2017), the authors reported 58% survival to discharge from hospital and 5-year survival of 46%. Major complications reported in their series were bleeding requiring reoperation/intervention (38%), renal failure requiring dialysis (46%), bacterial infections (37%), fungal infections (24%), critical limb ischaemia (10%), and stroke (13%). However, all these complications are associated with ECMO cannulation [43]. Complications associated with CentriMag pump are pump thrombosis and hemolysis and both the complications have rarely been reported. Furthermore, Impella results are inferior to CentriMag in terms of survival when used for supporting Interagency Registry for Mechanically Assisted Circulatory Support (INTERMACS) 1 patients. Also, Impella requires careful positioning and frequent confirmation of position across the aortic valve and usually does not provide full circulatory support [43].

### Extracorporeal membrane oxygenation

Veno-arterial extracorporeal membrane oxygenation (VA-ECMO) is an extension of CPB initially described in the 1970s by Hill and colleagues [46]. ECMO technology has evolved significantly and presently it can provide robust biventricular as well as respiratory support for days to weeks in patients with severe refractory CS and the patient being extubated and ambulant [47].

#### Components and cannulation

The VA-ECMO circuit consists of venous and arterial cannulas for drainage and return of blood, respectively, a hollow fiber membrane oxygenator for gas exchange, and a centrifugal pump for propelling the blood. The presence of a membrane oxygenator is a critical distinguishing feature of ECMO from other AMCS devices.

VA-ECMO can be established centrally or peripherally and percutaneously or surgically. For post-cardiotomy setting, central VA-ECMO is usually preferred, and oxygenated blood is returned directly into the ascending aorta, while, for other etiologies of CS, peripheral VA-ECMO is preferred and the femoral artery and vein are cannulated percutaneously. Alternate sites for cannulation are the AxA or SCA and internal jugular vein (IJV). The advantages of upper body cannulation include ease of ambulation, reduced risk of infection, limb ischemia, and cannula site bleeding. An appropriate-sized cannula should be selected to prevent vascular injury while achieving lower negative inflow (preferably <  − 50 mmHg) and lower outflow (< 300 mmHg) pressures [48]. In our center, when FA is cannulated for VA-ECMO, we always insert a 6-Fr distal reperfusion cannula into the superficial femoral artery to mitigate the risk of distal limb ischemia and splice it into the arterial limb of the circuit.

#### Indications

VA-ECMO is most commonly indicated as a short-term hemodynamic and respiratory support strategy for patients presenting with CS SCAI stage C–E and severe biventricular HF [42]. It can be instituted and initiated safely in a brief time in the cath lab, in the field (mobile ECMO programs), at the bedside, or in the operating room and even during ongoing cardiopulmonary resuscitation [49, 50]. Full VA-ECMO support allows time for the heart to recover and perform diagnostic and therapeutic interventions. Multiple clinical trials are currently ongoing with the aim to address the potential clinical benefits of early VA-ECMO initiation in various patient populations with CS [51].

#### Hemodynamic aspects of VA-ECMO support

For patients with CS, VA-ECMO has the highest capability to reduce the myocardial pressure–volume area (sum of myocardial potential energy and myocardial stroke work) irrespective of ventricular function. The myocardial pressure–volume area is further reduced by the weaning of inotropic and vasopressor drugs. All these prevent the vicious cycle of maladaptive neurohormonal and vascular mechanisms. Also, RV function is not critical VA-ECMO to provide systemic perfusion due to the absence of its reliance on transpulmonary flow.

VA-ECMO also improves tissue perfusion and reduces the generation of toxic metabolites especially in organs with portal circulation, such as the gastrointestinal tract, liver, and kidney by increasing the systemic arterio-venous pressure gradient (increase in MAP and reduction in CVP). Fluid removal can be enhanced by splicing a continuous veno-venous hemodialysis machine (CVVHD) into the VA-ECMO circuit [51–55].

#### LV distension during VA-ECMO

The Achilles heel of VA-ECMO is the distension of LV and it occurs in the setting of two co-existing conditions: (I) continuous source of blood to fill the LV and (II) inability of the LV to empty itself. During VA-ECMO, a significant amount of blood continuously returns to the LV from AR, Thebesian, and bronchial veins via the left atrium, and systemic venous return not captured by the venous cannula. Among all, uncaptured systemic venous return is the most significant source of LV filling, and it is directly proportional to the RV function. Also, due to the lack of reservoir, and longer and thinner peripheral venous cannulas with higher impedance, a significant amount of blood escapes drainage. Above this, retrograde aortic blood flow at high pressure increases the LV afterload. Therefore, to eject the returning blood, LV must have enough contractile function to overcome this afterload. In case of severe dysfunction, LV may be unable to generate enough pressure to overcome high transvalvular gradient and the aortic valve may remain closed throughout the cardiac cycle. This may result in increased LV wall stress, LVEDP, and myocardial oxygen demands as well as stasis of blood in the aortic root and LV with potential of thrombus formation. Furthermore, increased LVEDP results in elevated pulmonary venous pressures, leading to pulmonary edema and hemorrhage, and subsequent hypoxia. Risk of LV and aortic root thrombus formation is higher with peripheral cannulation due to a larger column of blood stasis and carries the risk of embolization to coronary, cerebral, and systemic vessels.

Therefore, patients with severe dysfunctional LV need LV venting during VA-ECMO support. In the presence of MR, the mitral valve serves as a “pop-off” for the LV at the expense of pulmonary edema. In a computer-simulated model of peripheral VA-ECMO by Marc Dickstein, LV distension and PCWP were inversely proportional to the LV contractility and directly proportional to the LV afterload [55–58].

#### Indications for LV venting and unloading

Indicators of good LV decompression on VA-ECMO are aortic valve opening and blood ejection with each cardiac contraction, systemic arterial pulse pressure > 10 mmHg, and PCWP < 18 mmHg. As an initial therapy, inotropes (dobutamine and milrinone), diuretics, CVVHD, and vasopressors are tried to maintain volume status and blood pressure. Additionally, ECMO flows can also be titrated to the lowest acceptable level to reduce the afterload that facilitates better ejection.

If despite optimal medical management, LV remains distended with stagnation of blood on echocardiography, PCWP is high, and the patient has refractory ventricular arrhythmias, LV venting or unloading should be considered [59]. Percutaneous transvenous atrial septostomy can be created under fluoroscopic and/or echocardiographic guidance to vent the LV. However, LV venting through atrial septostomy is passive, dependent upon associated MR, and limited by the pressure gradient between the atria.

## AMCS devices for unloading the left ventricle on VA-ECMO

### Intra-aortic balloon pump

The IABP is the weakest venting device that improves the LV emptying by decreasing afterload and relatively preserved LV function is an important pre-requisite. In a large, pooled analysis of 1517 patients by Cheng et al. in 2015, the authors reported no survival benefit with adding the IABP to VA ECMO [60]. However, in a recent retrospective study by Chen et al. from China involving 60 patients with post-cardiotomy CS who underwent concomitant IABP and ECMO placement, the authors reported concurrent implantation of ECMO with IABP result in improved survival to hospital discharge compared to subsequent placement of IABP (81% vs. 50%, *P* = 0.014). Furthermore, in their study, concurrent implantation of IABP with ECMO was an independent predictor of survival to discharge (OR = 0.177, *P* = 0.015, 95% CI: 0.044–0.718) [61]. In another retrospective study by Meani et al. involving 10 patients who underwent IABP placement for protracted aortic valve closure after peripheral VA-ECMO implantation, the authors reported that IABP placement effective in reversing the protracted aortic valve closure and unloading the LV. However, this improvement did not result in improvement in outcome of the patients (20% survival) [62]. A major drawback of all these studies is their retrospective nature. We believe that the role of IABP in LV unloading after VA-ECMO placement needs further exploration with prospective studies.

### Percutaneous vents

Left atrial vents can be placed after doing an atrial septostomy by trans-septal puncture, ballooning, and blade septostomy. Similarly, a pulmonary artery or retrograde trans-aortic catheter can be placed percutaneously [63]. All these vents are partially effective in unloading LV and efficiency depends significantly upon the size of cannula. TandemHeart is the most effective left atrial (LA) vent in reducing LV preload due to its active drainage. However, due to the expertise required for atrial septostomy and cannula placement, and high incidence of complications; TandemHeart is seldom used for this purpose.

### Impella

In patients with VA-ECMO with severe LV dysfunction and a well-functioning lung, Impella can be inserted for LV unloading called ECPella. Impella by directing blood away from heart decreases the LV preload without increasing afterload, and drainage by Impella is continuous and independent of arrhythmias. Furthermore, Impella improves CBF and prevents the aortic root stasis [64]. In our institute, we prefer VA-ECMO and Impella 5.5 placement through AxA over Y chimney graft and venous cannula through IJV. Advantages of our technique are ease of ambulation and weaning and ECMO decannulation with over-sewing the Y limb of the graft that can be done under local anesthesia and sedation [65]. Daily chest X-rays should be obtained to assess the pulmonary edema, and Impella and ECMO cannula position. Echocardiography should be performed to ensure the Impella position and LV decompression [55].

## Results

Studies by Patel et al., Tepper et al., and Pappalardo et al. have shown improved survival in patients supported with ECPella with reduced all-cause 30-day mortality compared to patients supported with VA-ECMO with inotropes or surgical LV venting. Furthermore, ECPella patients had a higher rate of successful bridging to either recovery or further therapy [66–68].

## Open surgical and minimally invasive LV unloading (Fig. 3)

**Fig. 3:**
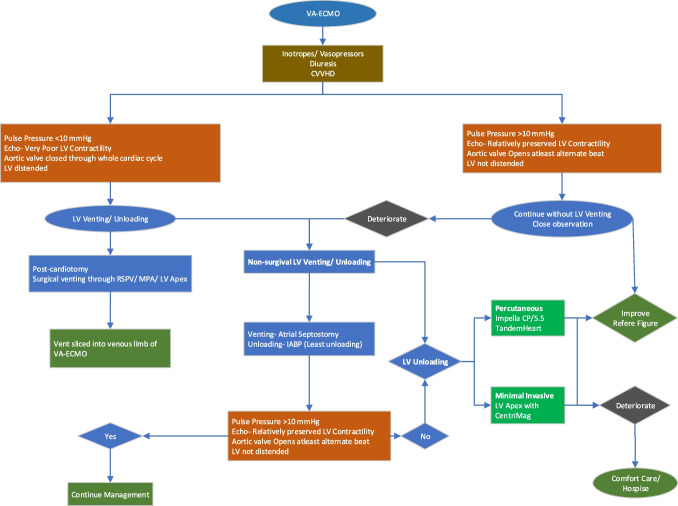
Left ventricular venting and unloading in a patient on VA-ECMO. VA-ECMO, veno-arterial extracorporeal membrane oxygenation; CVVHD, continuous veno-venous hemodialysis; echo, echocardiography; LV, left ventricle; RSPV, right superior pulmonary vein; MPA, main pulmonary artery; IABP, intra-aortic balloon pump

In patients with post-cardiotomy CS, vents can be placed surgically into the LV via the right superior pulmonary vein or via LV apex. In non-post-cardiotomy patients, a surgical vent can still be placed into the LV apex via small left anterolateral thoracotomy and sliced into the venous limb of the ECMO cannula using CentriMag pump (VA-ECMO + CentriMag) [55]. An adjustable tube clamp should be used to titrate the amount of drainage from each of the two inflow limbs (venous and LV apex). Compared to peripheral ECPella, this approach perfuses the oxygenated blood into the aortic root, brain, and upper body and it unloads both the ventricles more efficiently [55, 69].

### Weaning of VA-ECMO with or without LV unloading

During VA-ECMO support, recovery of ventricular systolic function manifests as improvement in the pulsatility with a gradual increase in systemic arterial pulse pressure, recovery of ventricular function on echocardiography, and reduction in pressor requirement to low level. After weaning the inotropes to minimum, if the patient remains stable with good pulsatility, absence of significant arrhythmias, and good ventricular function on echocardiography, ECMO flows are reduced 0.5–1 L every 30 min until ECMO flow is reduced to 1.5–2 L/min. If the patient remains stable, ECMO is turned off and the patient is decannulated. However, if the patient becomes unstable, ECMO flows are increased to 2 L/min or above and the patient should be investigated for the failure of weaning trial and optimized before another trial of ECMO wean after 24 h. In patients supported with ECPella, or VA-ECMO + CentriMag, Impella flow or drainage through LV vent is reduced first and then ECMO flow is reduced to 1.5–2 L/min. If the patient remains stable, VA-ECMO is discontinued and decannulated in patients with ECPella while and only venous cannula is removed in patients with VA-ECMO + CentriMag. Impella or LV apical vent is continued, and flow can be increased if additional support is required [55].

### Contraindication and complications of VA-ECMO

Severe PAD (percutaneous only) and moderate or more AR are absolute contraindications to VA-ECMO. Complications associated with ECMO are pump thrombosis, bleeding, limb ischemia, and Harlequin syndrome.

## Selection of AMCS device

### Unloading the LV in patients with AMI (Fig. 4)

**Fig. 4:**
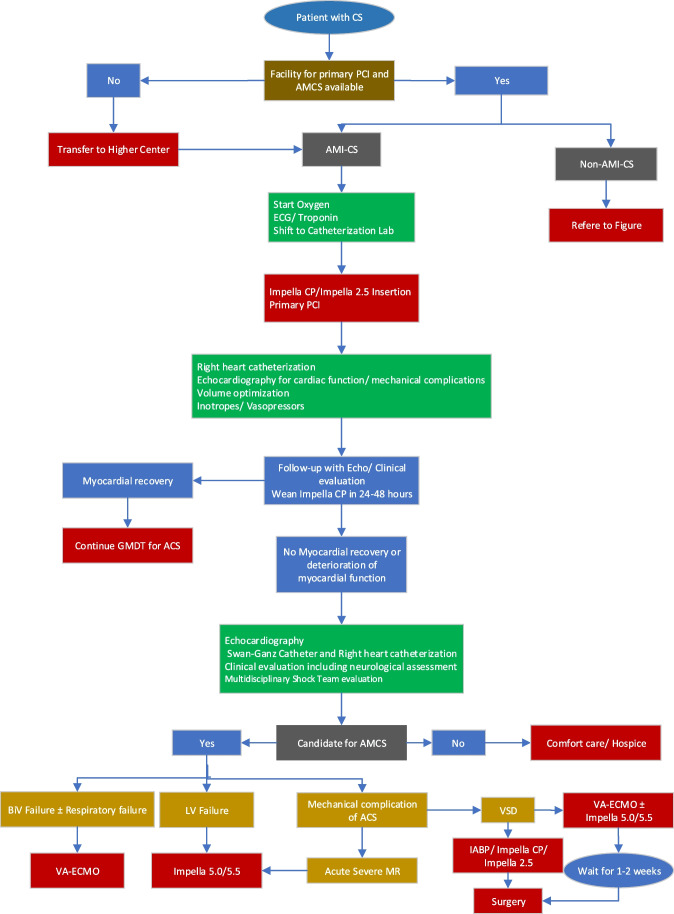
Decision making and choosing the AMCS in patients with AMI-CS and complications of AMI. CS, cardiogenic shock; AMI, acute myocardial infarction; ECG, electrocardiography; PCI, percutaneous coronary intervention; Echo, echocardiography; GMDT, guideline-directed medical therapy; LV, left ventricle; VSD, ventricular septal defect; BiV, biventricular; VA-ECMO, veno-arterial extracorporeal membrane oxygenation; MR, mitral regurgitation

In patients undergoing primary PCI for AMI, door-to-unloading time instead of door-to-needle time is gaining wide acceptance. Impella CP or Impella 2.5 insertion prior to PCI to unload the LV has shown to improve the myocardial salvage and periprocedural hemodynamic stabilization. Studies are ongoing to evaluate the outcome of Impella insertion prior to primary PCI [70].

### Complications of AMI (Fig. 4)

Acute ischemic MR and post-AMI VSD are particularly well-suited for AMCS devices as hemodynamic disturbance is usually acute and substantial. In patients with post-AMI-VSD, IABP may be optimal if the patient is wheeled into the operating room or the patient is hemodynamically and metabolically stable on minimal inotropes/vasopressors. However, if delayed surgical repair is contemplated, the patient is in SCAI stage C–E; Impella 5.5 or Impella 5.5 + Impella RP/Protek Duo/CentriMag or VA-ECMO ± LV unloading should be done to give complete rest to the LV and reducing the shunting across VSD. Furthermore, patients with AMI with severely depressed LV and RV infarction may also benefit from AMCS devices. Presently, IABP is the most commonly used device for AMI-related complications due to its ease of insertion and wide availability. However, early switching to appropriate and more robust AMCS device may improve patient outcome. In patients with severely depressed LV and acute severe ischemic MR, Impella 5.5,while for RV-AMI, Protek Duo, CentriMag, or Impella RP are optimal AMCS devices. The choice of the device should be guided by the type of AMI (LV-AMI vs. RV-AMI), severity of ventricular dysfunction, and size of VSD [6, 71].

### Severe heart failure in the setting of non-ischemic cardiomyopathy (Fig. 5)

**Fig. 5:**
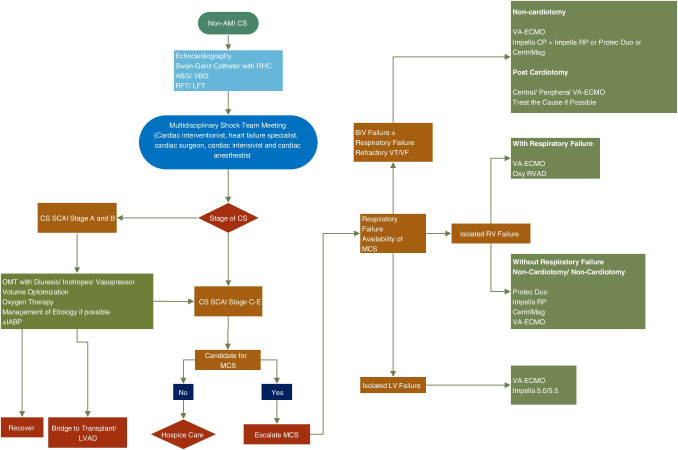
Decision making and choosing the AMCS in patients with non-AMI-CS. CS, cardiogenic shock; AMI, acute myocardial infarction; ABG, arterial blood gas; VBG, venous blood gas; RFT, renal function test; LFT, liver function test; SCAI, Society of Cardiovascular Angiography and Interventions; OMT, optimal medical therapy; LVOT, left ventricular assist device; VT, ventricular tachycardia; VF, ventricular fibrillation; oxy-RVAD, right ventricular assist device with oxygenator

In patients with ACHF as well as other acute cardiomyopathies, e.g., fulminant myocarditis, stress cardiomyopathy, peripartum cardiomyopathy, and toxic cardiomyopathies, AMCS devices may be used as a bridge to recovery, bridge to decision, or bridge to LVAD/ transplant. IABP may be suitable for patients presenting in SCAI stages A and B while more robust devices should be chosen for patients with SCAI stages C to E. The choice of the device should be guided by type (RV, LV, biventricular) and severity of ventricular dysfunction [6, 39–45, 72].

### Primary cardiac allograft dysfunction and post-transplant RV failure

Primary allograft dysfunction may occur due to acute cellular or antibody-mediated rejection, prolonged ischemia time, and inadequate organ preservation. Acute RV failure occurs due to pulmonary hypertension in the recipient, ischemia–reperfusion injury, and excess volume/blood product resuscitation. VA-ECMO with inotropes and inhaled nitric oxide (iNO) and other pulmonary vasodilator therapy provides time for the donor RV to recover [6, 73].

### Post-cardiotomy cardiogenic shock

Patients usually develop biventricular dysfunction and central or peripheral VA-ECMO with or without LV unloading is preferred as a bridge to recovery or bridge to decision. This also provides additional time to investigate the cause of CS. However, in patients with isolated RV or LV failure, a more specific AMCS device may be chosen as these are easier to manage, need less anticoagulation, and are less labor-intensive compared to VA-ECMO [6, 72]. Also, IABP or Impella can be placed preoperatively in patients undergoing high-risk CABG ± MV repair. In patients with multivessel coronary artery disease with or without left main coronary artery disease and have LV ejection fraction > 35%, IABP is sufficient support [74–76]. However, in patients with LV ejection fraction < 35% with or without moderate to severe MR, preoperative Impella insertion can decrease the incidence of post-cardiotomy shock, improve bypass graft survival, and lower PA pressures and mitral regurgitation [77].

### Refractory arrhythmias

For patients with recurrent arrhythmia, Impella is initial AMCS device of choice. However, for recurrent and refractory ventricular arrhythmias, VA-ECMO can provide adequate biventricular support [72].

### Prophylactic use for high-risk percutaneous procedures

Pre-procedure insertion of Impella CP or 2.5 is preferred in patients undergoing high-risk percutaneous intervention, e.g., PCI in severe LV dysfunction with ejection fraction < 20 to 30%, complex coronary artery disease involving large myocardial territory (sole-remaining vessel, left main or three-vessel disease), percutaneous valve intervention, and high-risk or complex ablation of ventricular tachycardia. By unloading the LV and improving coronary perfusion, arrhythmias can be tolerated for longer duration without fear of systemic hypoperfusion [6, 45, 72].

## Escalation and de-escalation of AMCS devices (Figs. 6 and 7)

**Fig. 6:**
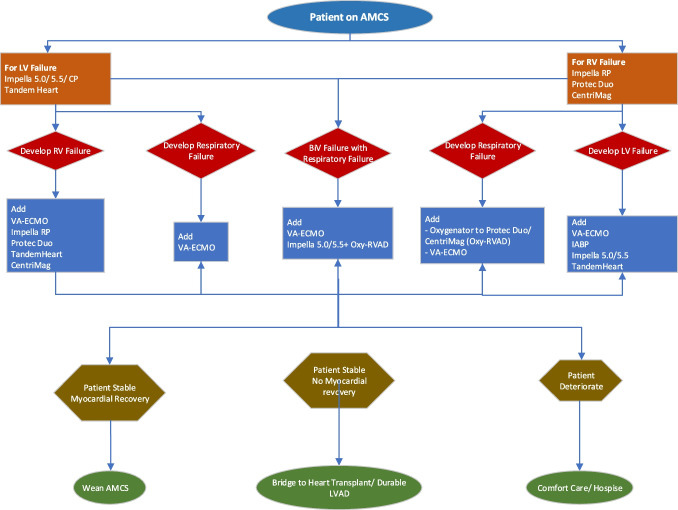
Decision making in escalation of AMCS device. AMCS, acute mechanical circulatory support; LV, left ventricle; RV, right ventricle; VA-ECMO, veno-arterial extracorporeal membrane oxygenation; IABP, intra-aortic balloon pump; oxy-RVAD, right ventricular assist device with oxygenator; LVAD, left ventricular assist device

**Fig. 7:**
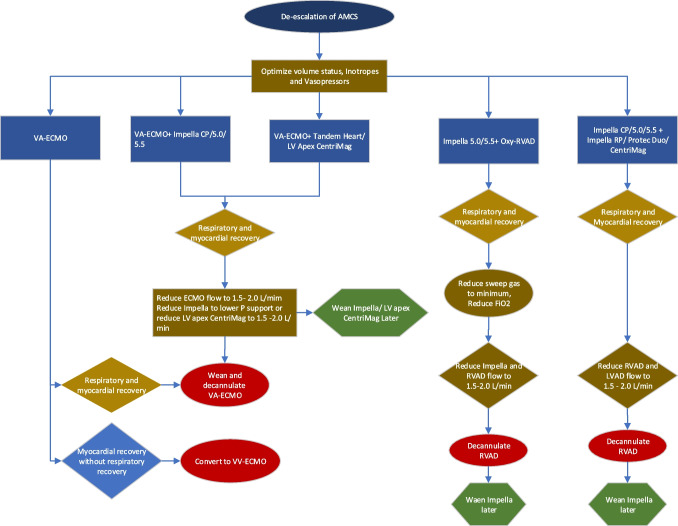
Decision making in de-escalation of AMCS devices. AMCS, acute mechanical circulatory support; LV, left ventricle; RV, right ventricle; VA-ECMO, veno-arterial extracorporeal membrane oxygenation; IABP, intra-aortic balloon pump; oxy-RVAD, right ventricular assist device

Any patient with AMCS device should be closely monitored with echocardiography, hemodynamics, and biochemical parameters including lactate, renal and liver function tests, and urine output. In patients with deterioration on AMCS, we prefer to escalate the AMCS early to prevent irreversible organ damage. Patients presenting with high lactate and multiorgan dysfunction are better managed with VA-ECMO or ECPella at the outset to facilitate organ recovery. Patients with lopsided AMCS devices may need additional contralateral AMCS devices or oxygenator for ventricular or respiratory failure, respectively. Patients who have no myocardial recovery even after escalation of AMCS should be bridged to LVAD or heart transplant. Patients who are not a candidate for either treatment (e.g., age > 70–75 years, multiple comorbidities, poor family support) should be redirected to comfort care or hospice.

Patients with lopsided AMCS and have myocardial recovery should undergo de-escalation of AMCS to minimal support and then removed. In patients with biventricular AMCS, after the myocardial recovery, RV-AMCS should be removed first continuing the LV support. If the patient remains stable, LV-AMCS should be weaned and removed. For VA-ECMO with a percutaneous or surgically placed LV unloading device, ECMO is weaned and removed first while continuing with the LV support and if the patient remains stable, LV unloading device can be removed. In patients with VA-ECMO without LV unloading, ECMO can be weaned and removed after myocardial recovery. However, for patients who have myocardial recovery without pulmonary improvement, VA-ECMO can be converted to veno-venous-ECMO [78, 79].

## Conclusion

Percutaneous and surgical AMCS devices for the management of cardiogenic shock have significantly evolved. They can be rapidly deployed in patients with varying etiologies and severities of cardiogenic shock. Device or combination of devices selection should be guided by patient factors, clinical setting, extent of cardiorespiratory support required, operator/institutional experience, and device-specific complications and risks. Multi-disciplinary team approach, careful patient and device selection, and complete understanding of treatment goal and hemodynamic consequences of AMCS devices can potentially improve the patient outcome.
